# Effects of intravenous lidocaine, dexmedetomidine, and their combination on IL-1, IL-6 and TNF-α in patients undergoing laparoscopic hysterectomy: a prospective, randomized controlled trial

**DOI:** 10.1186/s12871-020-01219-z

**Published:** 2021-01-06

**Authors:** Siqi Xu, Shenghong Hu, Xia Ju, Yuanhai Li, Qing Li, Shengbin Wang

**Affiliations:** 1 Department of Anesthesiology, The Affiliated Anqing Hospital of Anhui Medical University, Anqing, 246000 China; 2 Department of Anesthesiology, The First Affiliated Hospital of Anhui Medical University, Hefei, 230032 China; 3 Department of Gynaecology and Obstetrics, The Affiliated Anqing Hospital of Anhui Medical University, Anqing, 246000 China

**Keywords:** Lidocaine, Dexmedetomidine, Interleukin, Tumor necrosis factor-α, Laparoscopic hysterectomy

## Abstract

**Background:**

Surgical-related inflammatory responses have negative effects on postoperative recovery. Intravenous (IV) lidocaine and dexmedetomidine inhibits the inflammatory response. We investigated whether the co-administration of lidocaine and dexmedetomidine could further alleviate inflammatory responses compared with lidocaine or dexmedetomidine alone during laparoscopic hysterectomy.

**Methods:**

A total of 160 patients were randomly allocated into four groups following laparoscopic hysterectomy: the control group (group C) received normal saline, the lidocaine group (group L) received lidocaine (bolus infusion of 1.5 mg/kg over 10 min, 1.5 mg/kg/h continuous infusion), the dexmedetomidine group (group D) received dexmedetomidine (bolus infusion of 0.5 μg/kg over 10 min, 0.4 μg/kg/h continuous infusion), and the lidocaine plus dexmedetomidine group (group LD) received a combination of lidocaine (bolus infusion of 1.5 mg/kg over 10 min, 1.5 mg/kg/h continuous infusion) and dexmedetomidine (bolus infusion of 0.5 μg/kg over 10 min, 0.4 μg/kg/h continuous infusion). The levels of plasma interleukin-1 (IL-1), interleukin-6 (IL-6), and tumor necrosis factor-α (TNF-α) at different time points were the primary outcomes. Secondary outcomes included hemodynamic variables, postoperative visual analogue scale (VAS) scores, time to first flatus, and incidence of nausea and vomiting after surgery.

**Results:**

The levels of plasma IL-1, IL-6, and TNF-α were lower in groups D and LD than in group C and were lowest in group LD at the end of the procedure and 2 h after the operation (*P* < 0.05). The VAS scores were decreased in groups D and LD compared with group C (*P* < 0.05). The heart rate (HR) was decreased at the end of the procedure and 2 h after the operation in groups D and LD compared to groups C and L (*P* < 0.001). The mean blood pressure (MBP) was lower at 2 h after the operation in groups L, D, and LD than in group C (*P* < 0.001). There was a lower incidence of postoperative nausea and vomiting (PONV) in group LD than in group C (*P* < 0.05).

**Conclusions:**

The combination of lidocaine and dexmedetomidine significantly alleviated the inflammatory responses, decreased postoperative pain, and led to fewer PONV in patients undergoing laparoscopic hysterectomy.

**Trial registration:**

ClinicalTrials.gov (NCT03276533), registered on August 23, 2017.

## Background

Laparoscopic procedures are widely adopted for gynecological patients due to certain benefits, including decreased intensity of pain after surgery, improved postoperative recovery of intestinal function, and improved cosmetic effects [1]. However, tissue injury induced by surgical trauma stimulates the systemic inflammatory cascade to elicit the release of a large number of inflammatory cytokines [2]. High levels of inflammatory cytokines not only affect wound healing but may also be associated with a large number of complications, such as postoperative pain, fatigue, and cognitive dysfunction [3–5]. Dexmedetomidine, a highly selective alpha 2-adrenergic agonist, can lead to hypnosis, sedation, analgesia and minimal respiratory depression [6]. As an anesthetic adjuvant, in view of reducing catecholamine release [7], sparing opioids [8], and improving the quality of recovery during the anesthesia period [9, 10]_,_ dexmedetomidine has been widely used in the clinical setting. Animal and clinical studies have revealed that the systemic administration of dexmedetomidine may exert anti-inflammatory effects [11, 12]. The systemic administration of lidocaine has been increasingly used for surgical patients due to its potential beneficial effects, including opioid-sparing [13], analgesic [14] and anti-inflammatory properties [15]. Our previous study indicated that dexmedetomidine plus lidocaine infusion may further decrease the intensity of postoperative pain, lower the requirement of fentanyl after surgery, and accelerate bowel function recovery than lidocaine or dexmedetomidine infusion alone [16]. Although intravenous lidocaine and dexmedetomidine infusion alone may exert anti-inflammatory efficacy, the degree to which the combination of lidocaine and dexmedetomidine infusion inhibits the inflammatory response has not been evaluated. Therefore, we hypothesized that the co-administration of lidocaine and dexmedetomidine could further decrease the levels of plasma TNF-α, IL-6, and IL-1 compared with lidocaine and dexmedetomidine alone after laparoscopic hysterectomy.

## Methods

The research approach was approved by the Ethics Committee of Anqing Municipal Hospital and registered at www.clinicaltrials.gov (Number: NCT03276533, registration date: 08/23/2017). All methods were performed in accordance with the relevant guidelines and regulations in our present study. All subjects provided informed consent at least 12 h before surgery. The inclusion criteria of our trial included American Society of Anesthesiologists (ASA) physical status I and II, age between 40 and 65 years, and undergoing laparoscopic hysterectomy with general anesthesia. The exclusion criteria included a history of allergy to local anesthetics, preoperative atrioventricular block and bradycardia, impaired kidney or liver function, underlying severe respiratory disease, and a history of opioid use and psychiatric disease. Patients were randomized into four groups (groups L, D, LD, and C) by a nurse in the postanesthesia care unit (PACU) who did not participate in the study according to computer-generated random numbers and sealed envelopes. Patients in group L received a bolus infusion of lidocaine (2%; 1.5 mg/kg over 10 min before the induction of anesthesia), and then lidocaine was infused at a rate of 1.5 mg/kg/h, which was ceased 30 min before the end of the operation [17]. Patients in group D received a bolus infusion of dexmedetomidine (0.5 μg/kg over 10 min before the induction of anesthesia), and then dexmedetomidine was infused at a rate of 0.4 μg/kg/h, which was ceased 30 min before the end of operation [16]. Patients in group LD received a bolus infusion of lidocaine (2%; 1.5 mg/kg) and dexmedetomidine (0.5 μg/kg) over 10 min before the induction of anesthesia, and then lidocaine and dexmedetomidine were infused at a rate of 1.5 mg/kg/h and 0.4 μg/kg/h, respectively, which were ceased 30 min before the end of the operation. Patients in group C received the same volume of normal saline (40 mL) 10 min before the induction of anesthesia, and then normal saline (0.9%) was continuously infused in an equal volume (40 mL/h), and ceased 30 min before the end of the operation. Study participants, including anesthesiologists, clinicians, and subjects, were blinded to the treatment assignments. The drug solutions in each group were provided by a nurse in the PACU who did not participate in the trial.

The primary endpoints in our study included the levels of plasma IL-1, IL-6, and TNF-α at different time points. The secondary endpoints included intraoperative propofol and remifentanil consumption, HR, MBP, VAS scores, time to first flatus, incidence of nausea and vomiting after the operation, and rescue analgesics (fentanyl).

Basal vital signs, including MBP, peripheral oxygen saturation (SPO_2_), electrocardiogram (ECG), HR and pressure of end-tidal CO_2_ (PetCO_2_), were established for each patient. All subjects received Ringer’s lactate (4–6 mL/kg/h) for compensatory capacity after arriving at the operating room. To reserve sufficient oxygenation, before the induction of anesthesia, all patients were given continuously oxygen (100%) for 3 to 5 min via a facemask. The induction of general anesthesia in all four groups was implemented with a target-controlled infusion (TCI) of plasma remifentanil and propofol. The initial TCI level of plasma propofol was set as 3.0 μg/mL [18]_._ The initial TCI level of plasma remifentanil was set as 5.0 ng/mL [19] 3 min after propofol infusion, and then cis-atracurium (0.15 mg/kg) was administered intravenously. Mechanical ventilation was implemented with an anesthesia machine (Aespire View, Datex-Ohmeda, USA). Respiratory parameters were adjusted to set PetCO_2_ between 35 mmHg and 45 mmHg. To maintain muscle relaxation during the anesthesia period, a supplemental dose of cis-atracurium was injected intermittently. During the surgery, BIS values were maintained between 50 and 60 by adjusting the TCI concentrations of plasma propofol during the anesthesia period in all patients. The hemodynamic variables were maintained within 20% of the preoperative baseline values by adjusting the infusion plasma concentrations of propofol and remifentanil. When patients had MBP < 60 mmHg or HR < 50 beats/min, ephedrine (6 mg) or atropine (0.5 mg) was intravenously administered, respectively. Fentanyl (1 μg/kg) was given intravenously 30 min before the end of surgery to alleviate the intensity of pain after surgery, and patient-controlled intravenous analgesia (PCIA) with 0.3 μg/kg/h fentanyl (a total regimen of 100 ml) was connected to each patient to deliver a bolus of fentanyl (0.075 μg/kg) with a 15-min lockout interval. Propofol and remifentanil infusions were terminated at the end of the procedure, and ondansetron (0.1 mg/kg) was injected to prevent nausea and vomiting after the operation. Neostigmine (20 μg/kg) and atropine (10 μg/kg) were injected intravenously to reverse neuromuscular blockade when spontaneous respiration sufficiently recovered. The endotracheal tube was removed when the train-of-four (TOF) ratio was at least 0.9 and patients were able to open their eyes according to verbal instructions. The patients were transferred to the PACU by an anesthesiologist 5 min after the endotracheal tube was removed. All patients were observed for 2 h in the PACU. The surgical procedure was completed by the same operative team, and the target pressure of carbon dioxide (CO_2_) pneumoperitoneum was maintained between 10 mmHg and 12 mmHg during the perioperative period.

The levels of plasma IL-1, IL-6, and TNF-α were measured at different time points, including baseline, the end of surgery, and 2 and 24 h after the operation. Blood samples from each patient were placed in tubes and centrifuged within 30 min, and plasma was separated and stored at − 70 °C until analysis. Enzyme-linked immunosorbent assay kits (KANU BIOLOGICAL TECHNOLOGY CO., Ltd., Shanghai, China) were used to test the levels of cytokines.

A 10-cm visual analogue scale (VAS) was used to assess the intensity of pain after the operation during the first 24-h period (0 = no pain; 10 = most imaginable pain). A total of 25 μg of fentanyl was injected when the postoperative VAS score was > 3 and until the VAS score was ≤3.

The MBP and HR were recorded at baseline, at the end of surgery, and 2 h after the operation. Intraoperative propofol and remifentanil consumption, the operating time, the anesthesia time, VAS scores, the incidence of nausea and vomiting, rescue analgesics, and time to first flatus were recorded after surgery.

### Sample size calculation

Based on our pilot study, we chose the levels of plasma IL-1, IL-6, and TNF-α as the primary outcome. This study was powered to detect a difference in the plasma levels of IL-1, IL-6, and TNF-α among the four arms with a β value set at 20% and α value set at 5% from PASS software. The mean and SD values of plasma IL-1, IL-6, and TNF-α at the end of surgery in all four groups were as follows: $\overline{\mathrm{X}}$_Con_ = 2.6 pg/mL, $\overline{\mathrm{X}}$_Lido_ = 2.4 pg/mL, $\overline{\mathrm{X}}$_Dex_ = 2.3 pg/mL, $\overline{\mathrm{X}}$_Lido + Dex_ = 2.1 pg/mL, S_Con_ = 0.5 pg/mL, S_Lido_ = 0.5 pg/mL, S_Dex_ = 0.4 pg/mL, S_Lido + Dex_ = 0.3 pg/mL; $\overline{\mathrm{X}}$_Con_ = 22.2 pg/mL, $\overline{\mathrm{X}}$_Lido_ = 20.9 pg/mL, $\overline{\mathrm{X}}$_Dex_ = 19.0 pg/mL, $\overline{\mathrm{X}}$_Lido + Dex_ = 17.7 pg/mL, S_Con_ = 5.9 pg/mL, S_Lido_ = 5.3 pg/mL, S_Dex_ = 4.8 pg/mL, S_Lido + Dex_ = 4.7 pg/mL; and $\overline{\mathrm{X}}$_Con_ = 41.6 pg/mL, $\overline{\mathrm{X}}$_Lido_ = 39.9 pg/mL, $\overline{\mathrm{X}}$_Dex_ = 37.8 pg/mL, $\overline{\mathrm{X}}$_Lido + Dex_ = 32.9 pg/mL, S_Con_ = 7.2 pg/mL, S_Lido_ = 8.4 pg/mL, S_Dex_ = 8.9 pg/mL, S_Lido + Dex_ = 7.9 pg/mL. Therefore, 32, 33, and 22 subjects for each group were respectively obtained, and considering a possible 20% dropout rate, we ultimately intended to recruit a total of 40 subjects for each arm.

### Statistical analysis

We used SPSS v.17 (IBM Corp., Armonk, NY, USA) software to complete the statistical analyses in the present study. Data are expressed as the number or mean ± standard deviation. The χ^2^ test or Fisher’s exact test, as appropriate, was used for categorical data analysis. One-way analysis of variance (ANOVA) was used for continuous data analysis in all four groups. Repeated measures design analysis of variance was applied to compare differences in plasma IL-1, IL-6, and TNF-α and MBP and HR at different time points in all four groups. If group differences were found by ANOVA to be significant, Tukey’s *post- hoc* test was performed for further analysis. Statistical significance was defined as a *P* value < 0.05.

## Results

**Fig. 1:**
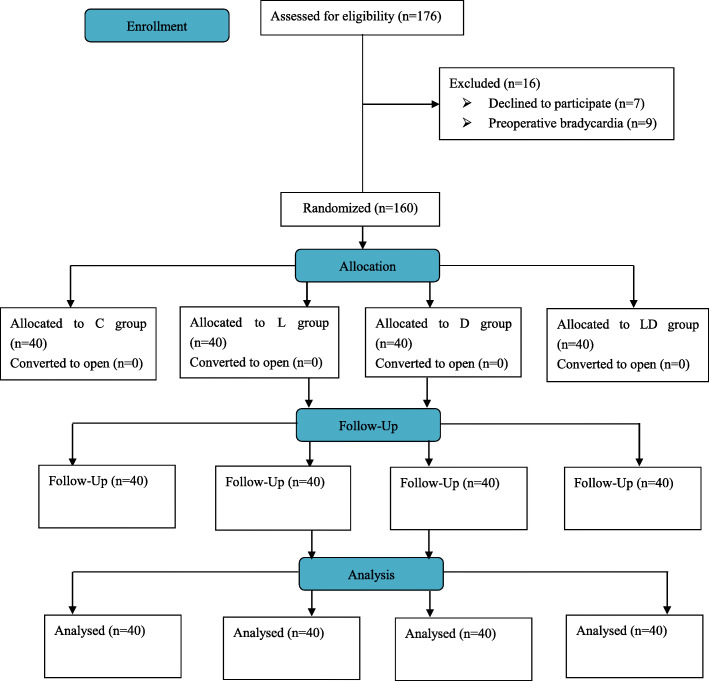
CONSORT flow diagram for the study

A total of 176 subjects were recruited for our trial, and sixteen patients were excluded, (nine patients with a history of preoperative bradycardia and seven patients who did not agree to participate in the study). Eventually, 160 subjects completed the present study. Data obtained from forty subjects in each arm were analysed (Fig. 1).

No significant differences were observed in any of the four arms in regard to ASA physical status, age, BMI, duration of the operation, weight, or duration of anesthesia (Table 1).

**Table 1:** Characteristics of patients

Variable	Group C (***n*** = 40)	Group L (***n*** = 40)	Group D (***n*** = 40)	Group LD (***n*** = 40)	***P*** value
Age (years)	47.2 ± 4.8	48.1 ± 5.6	47.8 ± 4.9	47.3 ± 5.4	0.848
Weight (kg)	59.9 ± 6.7	59.2 ± 6.3	57.8 ± 5.7	58.9 ± 6.2	0.541
BMI	24.6 ± 2.6	24.3 ± 2.3	23.8 ± 2.4	24.0 ± 2.2	0.402
Anesthesia time (min)	118.3 ± 9.2	120.7 ± 7.2	118.6 ± 7.7	120.2 ± 7.9	0.478
Operation time (min)	99.6 ± 10.4	101.0 ± 11.1	98.9 ± 8.5	100.2 ± 11.5	0.827
ASA physical status (I / II)	21/19	18/22	23/17	20/20	0.729

### Intraoperative consumption of remifentanil and propofol

The consumption of remifentanil and propofol was significantly decreased in groups L, D, and LD compared to group C (all *P* < 0.001). The consumption of remifentanil and propofol was lowest in group LD during the intraoperative period (*P* < 0.001). There were no significant differences in the intraoperative consumption of propofol and remifentanil between groups L and D (*P* = 0.740 and *P* = 0.097) (Table 2).

**Table 2:** Doses of propofol, remifentanil, rescue anesthetic, first flatus time, and incidence of PONV

Index	Group C (***n*** = 40)	Group L (***n*** = 40)	Group D (***n*** = 40)	Group LD (***n*** = 40)	***P*** value
Propofol dose (mg)	702.6 ± 56.3	650.3 ± 46.8^*^	640.0 ± 39.0^*^	554.0 ± 35.3^*#★^	< 0.001
Remifentanil dose (microg)	965.4 ± 87.7	835.8 ± 59.3^*^	799.2 ± 68.9^*^	550.6 ± 62.1^*#★^	< 0.001
Rescue anesthetic (microg)	33.8 ± 22.3	21.3 ± 19.2^*^	0.0 ± 0.0^*#^	0.0 ± 0.0^*#^	< 0.001
First flatus time (hours)	23.1 ± 3.8	20.4 ± 3.3^*★^	22.8 ± 3.4	19.9 ± 3.4^*★^	< 0.001
PONV (percentage)	21 (52.5)	17 (42.5)	13 (32.5)	10 (25)^*^	0.036

Data are presented as mean ± standard deviation (SD)

*C* control, *L* lidocaine, *D* dexmedetomidine, *LD* lidocaine and dexmedetomidine combination

^*^P < 0.05 versus group C, ^#^*P* < 0.05 versus group L, ^★^*P* < 0.05 versus group D

### Postoperative VAS scores at rest

The VAS scores in groups L, D, and LD were significantly decreased compared to those in group C at 2 and 6 h after the operation (*P* = 0.022, *P* = 0.028, *P* < 0.001, *P* < 0.001, *P* < 0.001, and *P* < 0.001, respectively). The VAS scores in group LD were lower than those in the other three groups at 2, 6, and 12 h after surgery (*P* < 0.01). There were no significant differences in postoperative VAS scores between groups L and D (*P* = 0.054, *P* = 0.168, *P* = 0.144, *P* = 0.839, respectively) (Table 3).

**Table 3:** VAS scores at rest during the first 24 h after operation

VAS scores	Group C (***n*** = 40)	Group L (***n*** = 40)	Group D (***n*** = 40)	Group LD (***n*** = 40)	***P*** value
2 h after surgery	3.3 ± 0.7	2.9 ± 0.6^*^	2.5 ± 0.6^*^	1.8 ± 0.6^*#★^	< 0.001
6 h after surgery	3.4 ± 0.8	3.0 ± 0.8^*^	2.6 ± 0.7^*^	1.7 ± 0.6^*#★^	< 0.001
12 h after surgery	2.7 ± 0.6	2.5 ± 0.8	2.2 ± 0.8^*^	1.6 ± 0.7^*#★^	< 0.001
24 h after surgery	2.0 ± 0.7	1.7 ± 0.7	1.5 ± 0.6^*^	1.3 ± 0.6^*#^	< 0.001

Data are presented as mean ± standard deviation (SD)

*C* control, *L* lidocaine, *D* dexmedetomidine, *LD* lidocaine and dexmedetomidine combination

^*^P < 0.05 versus group C, ^#^*P* < 0.05 versus group L, ^★^*P* < 0.05 versus group D

### Time to first flatus

The time to first flatus in groups L and LD was shorter than that in groups C and D (*P* = 0.005, *P* = 0.012, *P* < 0.001, and *P* = 0.001, respectively). There was no significant differences in the time to first flatus between groups C and D (*P* = 0.992) (Table 2).

### Incidence of nausea and vomiting after the operation

The number of patients who experienced nausea and vomiting in group LD (25%) was less than that in group C (52.5%) during the first 24 h after the operation (*P* = 0.012). There were no differences with respect to PONV in groups L (42.5%) and D (32.5%) compared to group C (52.5%) (*P* = 0.370 and *P* = 0.070). The incidence of PONV was lowest in group LD (Table 2).

### The levels of plasma TNF-α, IL-6, and IL-1 at different time points

The levels of plasma TNF-α, IL-6, and IL-1 were not significantly different between the four groups at baseline. Compared to baseline, the levels of plasma TNF-α, IL-6, and IL-1 were significantly elevated at the end of the operation and 2 h after surgery (all *P* < 0.001). Compared to group C, the levels of plasma TNF-α, IL-6, and IL-1 were significantly decreased at the end of the operation and 2 h after surgery in groups D and LD (all *P* < 0.05). The levels of plasma TNF-α, IL-6, and IL-1 were lower at the end of the operation and 2 h after surgery in group LD than in groups L and D (all *P* < 0.05). The levels of plasma TNF-α, IL-6, and IL-1 were not significantly different during the perioperative period or during the first 24 h after surgery between groups L and D. The levels of plasma TNF-α, IL-6, and IL-1 were not significantly different at 24 h after surgery in all four groups (Table 4).

**Table 4:** Comparison of plasma IL-1, IL-6, and TNF-α levels at different time points

Cytokines	Groups	T_**0**_	T_**1**_	T_**2**_	T_**3**_
IL-1(pg/ml)	Group C	1.27 ± 0.35	2.72 ± 0.56^△^	3.18 ± 0.57^△^	1.53 ± 0.36
	Group L	1.36 ± 0.27	2.56 ± 0.52^△^	2.97 ± 0.53^△^	1.51 ± 0.31
	Group D	1.38 ± 0.33	2.41 ± 0.48^△^	2.83 ± 0.59^△^	1.55 ± 0.37
	Group LD	1.35 ± 0.36	2.02 ± 0.45^△^	2.36 ± 0.47^△^	1.56 ± 0.34
	^a^*P*	0.599	0.519	0.290	0.907
	^b^*P*	0.431	0.035	0.030	0.396
	^c^*P*	0.744	< 0.001	< 0.001	0.221
	^d^*P*	0.993	0.519	0.656	0.805
	^e^*P*	0.995	< 0.001	< 0.001	0.594
	^f^*P*	0.958	0.04	0.001	0.985
IL-6 (pg/ml)	Group C	11.33 ± 2.57	23.98 ± 6.37^△^	26.05 ± 7.26^△^	12.65 ± 3.18
	Group L	10.95 ± 2.46	23.03 ± 5.87^△^	23.18 ± 5.07^△^	12.00 ± 3.11
	Group D	10.40 ± 2.94	20.60 ± 5.03^△^	21.75 ± 4.91^△^	11.43 ± 3.34
	Group LD	10.65 ± 2.66	17.15 ± 4.82^△^	18.30 ± 5.31^△^	11.18 ± 3.11
	^a^*P*	0.992	0.870	0.115	0.798
	^b^*P*	0.408	0.037	0.005	0.317
	^c^*P*	0.669	< 0.001	< 0.001	0.168
	^d^*P*	0.792	0.211	0.681	0.851
	^e^*P*	0.958	< 0.001	0.001	0.654
	^f^*P*	0.975	0.031	0.038	0.985
TNF-α (pg/ml)	Group C	12.55 ± 2.21	44.28 ± 6.89^△^	76.10 ± 10.65^△^	14.78 ± 3.49^△^
	Group L	12.80 ± 2.28	41.18 ± 8.55^△^	73.13 ± 9.31^△^	14.30 ± 2.66
	Group D	12.93 ± 2.35	38.80 ± 9.84^△^	69.45 ± 8.75^△^	13.80 ± 2.96
	Group LD	13.13 ± 2.64	33.78 ± 6.92^△^	62.95 ± 11.33^△^	13.43 ± 3.53
	^a^*P*	0.965	0.326	0.550	0.909
	^b^*P*	0.894	0.016	0.019	0.520
	^c^*P*	0.700	< 0.001	< 0.001	0.233
	^d^*P*	0.995	0.562	0.363	0.896
	^e^*P*	0.928	< 0.001	< 0.001	0.609
	^f^*P*	0.982	0.032	0.023	0.952

Data are presented as mean ± standard deviation (SD)

*T*_*0*_ baseline, *T*_*1*_ at the end of surgery, *T*_*2*_ 2 h after surgery, *T*_*3*_ 24 h after surgery, *C* control, *L* lidocaine, *D* dexmedetomidine, *LD* lidocaine and dexmedetomidine combination

^△^*P* < 0.05 compared with T_0_, ^a^*P* for group C versus group L, ^b^*P* for group C versus group D, ^c^*P* for group C versus group LD. ^d^*P* for group L versus group D, ^e^*P* for group L versus group LD, ^f^*P* for group D versus group LD

### MBP and HR at different time points

The MBP was significantly decreased in groups L, D, and LD at 2 h after surgery compared with group C (*P* = 0.034, *P* = 0.005, and *P* < 0.001, respectively). Compared with group L, the MBP was significantly lower at 2 h after surgery in group LD (*P* = 0.044). HR was significantly lower in groups D and LD at the end of surgery and 2 h after surgery than in groups C and L (all *P* < 0.001) (Table 5).

**Table 5:** Comparison of MAP and HR at different time points

Variables	Groups	T_**0**_	T_**1**_	T_**2**_
MAP (mmHg)	Group C	79.2 ± 8.2	73.9 ± 4.3^△^	77.1 ± 5.1
	Group L	78.6 ± 8.5	73.3 ± 5.1^△^	74.2 ± 4.7^△*^
	Group D	81.4 ± 7.1	72.2 ± 5.9^△^	73.6 ± 4.4^△*^
	Group LD	80.1 ± 6.2	71.1 ± 4.5^△^	71.5 ± 4.6^△*#^
HR (bpm)	Group C	74.3 ± 9.0	66.9 ± 6.3^△^	71.8 ± 5.7
	Group L	72.6 ± 8.5	66.3 ± 6.6^△^	68.6 ± 7.7
	Group D	74.8 ± 9.1	60.4 ± 5.2^△*#^	60.9 ± 5.4^△*#^
	Group LD	73.8 ± 9.2	60.0 ± 5.0^△*#^	60.2 ± 4.8^△*#^

Data are presented as mean ± standard deviation (SD)

*T*_*0*_ baseline, *T*_*1*_ at the end of surgery, *T*_*2*_ 2 h after surgery, *C* control, *L* lidocaine, *D* dexmedetomidine, *LD* lidocaine and dexmedetomidine combination

^△^*P* < 0.05 compared with T_0_, ^*^P < 0.05 versus group C, ^#^*P* < 0.05 versus group L

## Discussion

A significant finding from our trial was that the intraoperative combination of lidocaine and dexmedetomidine infusion further reduced inflammatory responses compared with lidocaine or dexmedetomidine infusion alone in patients following laparoscopic hysterectomy. Patients who received lidocaine plus dexmedetomidine infusion were associated with lower levels of plasma IL-1, IL-6, and TNF-α at the end of the operation and 2 h after surgery, lower VAS scores after surgery, and less intraoperative consumption of remifentanil and propofol compared with patients who received lidocaine or dexmedetomidine infusion alone. The co-administration of lidocaine and dexmedetomidine also resulted in a lower incidence of PONV.

Surgical-related tissue damage induces stress responses in the body, and further promotes the release of perioperative inflammatory cytokines, including IL-1, IL-6, and TNF-α [20]. The harmful inflammatory responses caused by surgical procedures have negative effects on postoperative outcomes in surgical patients and increase morbidity and mortality. The suppression of perioperative inflammatory responses is associated with less postoperative pain and improves postoperative outcomes. Therefore, it is important to effectively alleviate perioperative inflammatory responses for patients following surgery, especially major surgery. Animal experiments suggest that dexmedetomidine administration exerts some degree of protection for organs such as the lung [21], kidney [22], and brain [23]. The effects are associated with the anti-inflammatory property of dexmedetomidine. Kang et al. [24] found that dexmedetomidine reduced the levels of IL-1β and TNF-α at the end of peritoneal closure and 1 h after the operation in patients undergoing laparoscopic cholecystectomy. Dong et al. [25] revealed that systemic dexmedetomidine infusion decreased the levels of IL-1, IL-6, TNF-α, and C-reactive protein (CRP) at 1 h before the end of surgery and 24 h after surgery. The results of the present study indicated that dexmedetomidine infusion resulted in lower plasma IL-1, IL-6, and TNF-α concentrations at the end of the operation and 2 h after surgery as well as lower VAS scores at 2, 6, 12 and 24 h after surgery compared with normal saline infusion. Furthermore, the infusion of lidocaine combined with dexmedetomidine significantly decreased the levels of plasma IL-1, IL-6, and TNF-α at the end of the operation and 2 h after surgery and alleviated pain at 2, 6, and 12 h compared to the infusion of dexmedetomidine alone. This suggests that lidocaine plus dexmedetomidine infusion further suppresses the secretion of inflammatory cytokines and improves the postoperative intensity of pain compared with dexmedetomidine infusion alone and that postoperative pain relief may be associated with lower levels of plasma IL-1, IL-6, and TNF-α. Anti-inflammatory and analgesic effects of combination regimen decrease postoperative pain intensity and requirement of opioids, which reduce adverse effects associated with opioids, including PONV, delayed recovery of intestinal function, etc. Furthermore, it may be decrease cost and time of hospital and improve patient satisfaction in the clinical practice.

Lidocaine, an amide local anesthetic, is used for local anesthesia and to treat ventricular arrhythmias in the clinical setting. Currently, clinical studies have shown that intravenous lidocaine administration decreases opioid consumption [26] and postoperative pain [27] and accelerates bowel function recovery [28]. Sridhar et al. [29] showed that intravenous lidocaine was associated with low levels of CRP and IL-6 during the post-operative period following selective open abdominal surgeries. Song et al. [30] revealed that intravenous lidocaine infusion attenuated the initiation of an excessive inflammatory response during laparoscopic surgery and was associated with low levels of serum IL-6 and IL-8. Our results demonstrated that intravenous lidocaine did not significantly decrease the levels of plasma IL-1, IL-6, and TNF-α compared to intravenous normal saline. The cause of the inconsistent results may be associated with the dosage of lidocaine, type of surgery, and duration of continuous lidocaine infusion. The levels of plasma IL-1, IL-6, and TNF-α in group LD were lower than those in groups L and D. This suggested that the combination of lidocaine and dexmedetomidine infusion further alleviated inflammatory responses resulting from surgical trauma compared with lidocaine or dexmedetomidine infusion alone. This effect is attributed to following factors. (1) Lidocaine combined with dexmedetomidine may further attenuate the surgical stress response. (2) Lidocaine and dexmedetomidine may exert anti-inflammatory properties by different mechanisms of action. (3) The combination of lidocaine and dexmedetomidine infusion may exert additive anti-inflammatory effects. Our results also showed that lidocaine infusion was associated with lower VAS scores at 2 and 6 h after surgery, and a shorter time to first flatus. This finding suggests that lidocaine infusion may decrease early postoperative pain and facilitate faster bowel function in patients undergoing laparoscopic hysterectomy.

As an adjuvant drug, dexmedetomidine has been related to attenuating the MBP and HR and the response to surgical procedures. Several studies have demonstrated that the most common side effect of dexmedetomidine administration is bradycardia, which may or may not be accompanied by a transient increase in MAP [31–33]. Hence, we selected a smaller dose (0.5 μg/kg loading, 0.4 μg/kg/h infusion) in the present study to decrease adverse effects, including bradycardia, hypertension, and hypotension, and to avoid delayed recovery after the operation. Hemodynamic variables were stable at 2 h after surgery in groups D and LD. HR significantly decreased in groups D and LD compared to group C at the end of the operation and 2 h after surgery. Although lidocaine plus dexmedetomidine infusion and dexmedetomidine infusion alone increased the incidence of bradycardia in the present study, we found that HR < 50 bpm rarely occurred during the intraoperative period or during the PACU stay period after surgery.

PONV is prevalent in patients following gynecological laparoscopic surgery. The present study showed that patients who received lidocaine plus dexmedetomidine had a lower incidence of PONV than those receiving normal saline. The possible reasons include lower postoperative pain intensity and inflammatory cytokines.

Our study had several limitations. On the one hand, we only recorded a few inflammatory cytokines, including IL-1, IL-6, and TNF-α, and did not perform clinical measurements associated with inflammatory responses. On the other hand, we only observed the levels of plasma IL-1, IL-6, and TNF-α at the end of surgery and 2 and 24 h after surgery. These time points may not effectively reflect the levels of plasma IL-1, IL-6, and TNF-α caused by surgical insult in a time–dependent manner. Finally, we only focused on the intraoperative effects of lidocaine combined with dexmedetomidine following laparoscopic hysterectomy. This was a short study and lacked recovery profile assessments, such as patient satisfaction.

## Conclusions

The intraoperative combination of lidocaine and dexmedetomidine infusion further alleviated inflammatory responses, decreased postoperative pain, and led to fewer PONV in patients undergoing laparoscopic hysterectomy than either drug alone. Moreover, the improvement in postoperative pain and PONV may be associated with the suppression of inflammatory cytokines.

### The future perspectives

The development of ERAS and minimization of opioid or free opioid use may improve the recovery quality of patients. The inflammatory response may be associated with perioperative neurocognitive disorders (PNDs). The co-administration of lidocaine and dexmedetomidine may provide better anti-inflammatory and analgesic effects than lidocaine or dexmedetomidine alone. Therefore, the effects of the combination regimen on PNDs and the feasibility of the method for minimizing opioid or free opioid use were the points of concern.

## Data Availability

The datasets used and/or analysed during the current study are available from the corresponding author on reasonable request.

## Abbreviations

IV: Intravenous
ASA: American Society of Anesthesiologists
IL-1: Interleukin-1
IL-6: Interleukin-6
TNF-α: Tumor necrosis factor-α
MBP: Mean blood pressure
HR: Heart rate
CO_2_: Carbon dioxide
PONV: Postoperative nausea and vomiting
PACU: Postanesthesia care unit
SPO_2_: Peripheral pulse oximeter
ECG: Electrocardiogram
PetCO_2_: Pressure of end-tidal CO_2_
TCI: Target-controlled infusion
PCIA: Patient-controlled intravenous analgesia
TOF: Train of four
VAS: Visual analogue scale
SD: Standard deviation
ANOVA: One-way analysis of variance
CRP: C-reactive protein
MAP: Mean arterial pressure
